# Effect of Charge accumulation on Magnetic Rayleigh-Taylor Instability

**DOI:** 10.1038/s41598-019-47550-5

**Published:** 2019-07-29

**Authors:** Kangkang Liu

**Affiliations:** 1 Key Laboratory of Earth and Planetary Physics, Institute of Geology and Geophysics, Chinese Academy of Sciences, Beijing, China; 2 University of Chinese Academy of Science, Beijing, China

**Keywords:** Magnetospheric physics, Fluid dynamics

## Abstract

The intuitive physical description of magnetic Rayleigh-Taylor instability in some textbooks is generally considered to be: a small perturbation causes current discontinuity, which produce charge accumulation, the electric field produced by the accumulated charge amplify the initial perturbation. However, in calculating the linear growth rate of magnetic Rayleigh-Taylor instability (MRTI), the displacement current term in the Maxwell’s equations is ignored, which means the contribution of charge accumulation to the growth of MRTI is totally ignored. In this article, we calculated the linear growth rate of MRTI with the displacement current term in Maxwell’s equations retained. We show that the contribution of charge accumulation to the growth of MRTI is negligible only when the nominal Alfvén speed is much smaller than the light speed. For space plasma whose nominal Alfvén speed is generally much smaller than the light speed, the linear growth rate previous calculated is right but the intuitive physical description of MRTI is wrong. For laboratory plasma whose nominal Alfvén speed maybe comparable to light speed, the intuitive physical description of MRTI is also inaccurate and the linear growth rate of MRTI is undervalued.

## Introduction

When a heavier fluid is supported by a lighter fluid against gravity, the equilibrium is unstable to any perturbations of the interface. For if a parcel of heavier fluid is displaced downward with an equal volume of lighter fluid displaced upwards, the potential energy of the configuration is lower than that in the initial state, and the process goes on. This instability is called the Rayleigh-Taylor Instability (RTI)^1^. In addition to neutral fluids, RTI also plays an important role in space and laboratory plasmas^2–6^.

**Figure 1:**
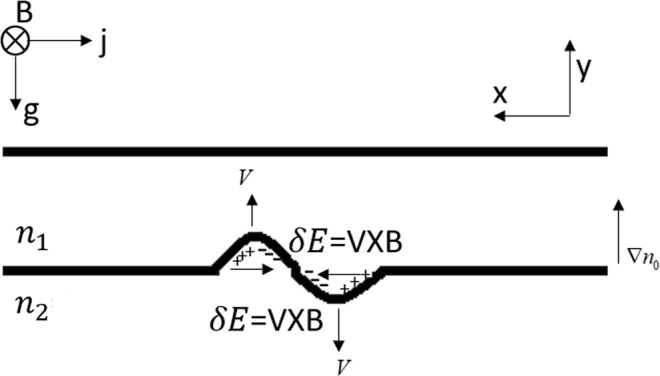
Schematic diagram of the MRTI.

For the neutral fluid, the momentum equation is $\frac{\partial (\rho {\bf{V}})}{\partial t}=\rho {\bf{g}}-\nabla p$, where *ρ*, **V**, **g**, *p* is the plasma density, the plasma bulk velocity, the gravity acceleration and the thermal pressure, respectively. In equilibrium state *ρ***g** = ∇*p*. The heavier fluids are supported by lighter fluids through pressure gradient in equilibrium state. For plasma in magnetic field, the momentum equation is $\frac{\partial (\rho {\bf{V}})}{\partial t}=\frac{1}{c}{\bf{J}}\times {\bf{B}}+\rho {\bf{g}}-\nabla p$, where c, **J**, **B** is the light speed, electric current and magnetic field strength, respectively. In equilibrium state $\rho {\bf{g}}=\nabla p-\frac{1}{c}{\bf{J}}\times {\bf{B}}$. The heavier fluids are supported by lighter fluids through pressure gradient and the **J** × **B** force in equilibrium state. The intuitive physical description of magnetic Rayleigh-Taylor instability (MRTI) in some textbooks^7^ is generally considered to be (Fig. 1): In equilibrium state a net current flows in in the horizontal direction and the current is proportional to the plasma density. There is thus a divergence, and charge will pile up on the edges of the small initial perturbation (charge accumulation). As a result, perturbation electric fields build up in the directions shown. These fields in turn cause an upward (downward) drift in the region where the density is low (high). Lower (higher) density plasma is therefore advected upward (downward), creating a larger perturbation, and the system is unstable. In the above physical description charge accumulation is the cause for the growth of RTI. However, in calculating the linear growth rate of magnetic Rayleigh-Taylor instability (MRTI), the displacement current term in the Maxwell’s equations is ignored^8,9^. When the displacement current term in the Maxwell’s equations is ignored, take the divergence of the Ampère’s circuital law without Maxwell’s addition shows that the current is always continuous. Which means the contribution of charge accumulation to the growth of MRTI is totally ignored. In this paper, we derived the expression of the linear growth rate of MRTI when charge accumulation is considered and discussed under what circumstances the displacement current can be ignored.

Assume a bundle of stratified plasma *ρ* = *ρ*(y) immersed in a magnetic field **B** (0, 0, B) and a gravity field **g** (0, −g, 0) (Fig. 1). To analyze the instability of the system, the relevant magnetohydrodynamic (MHD) equations and Maxwell’s equations written in Gaussian unit are:

1 $$\frac{\partial (\rho {\bf{V}})}{\partial t}=\frac{1}{c}{\bf{J}}\times {\bf{B}}+\rho {\bf{g}}-\nabla p$$

2 $$\frac{\partial \rho }{\partial t}+\nabla \cdot (\rho {\bf{V}})=0$$

3 $$\frac{\partial {\bf{E}}}{\partial t}=-\,4\pi {\bf{J}}+c\nabla \times {\bf{B}}$$

4 $$\nabla \cdot {\bf{B}}=0$$

5 $$\nabla \cdot {\bf{E}}=4\pi {\rho }_{c}$$

6 $$\nabla \cdot {\bf{J}}=-\,\frac{\partial {\rho }_{c}}{\partial t}$$

where **V**, *ρ*, c, **J**, **B**, p, **E**, *ρ*_*c*_ are the velocity of fluid element, plasma mass density, light speed, electric current density, magnetic field, thermal pressure, electric field, charge density, respectively.

The following derivation process of the linear growth rate of stratified plasma in a horizontal magnetic field in this manuscript was similar to that of Sharma *et al*.^10^ and Wu *et al*.^11^, except that charge conservation equation (Eq. (6)) instead of the current continuity equation was used in the derivation process. Form the momentum equation (Eq. (1)), one can see that the three term on the right side of the equation was equivalent. If charge accumulation effects considered, the **J** × **B** term will affect the linear growth rate of magnetic Rayleigh-Taylor instability.

To examine the stability of the system, we assume the following perturbation in physical quantities

$$\begin{array}{c}\rho ={\rho }^{0}+{\rho }^{1}\,p={p}^{0}+{p}^{1}\,{\bf{B}}={{\bf{B}}}^{0}+{{\bf{B}}}^{1}\,{\bf{J}}={{\bf{J}}}^{0}+{{\bf{J}}}^{1}\,{\bf{V}}={{\bf{V}}}^{0}+{{\bf{V}}}^{1}{{\bf{V}}}^{0}=0\\ {\bf{E}}={{\bf{E}}}^{0}+{{\bf{E}}}^{1}\,{{\bf{E}}}^{0}=0.\end{array}$$

In the above equations, quantities with superscripts 0 and 1 denote equilibrium and perturbation quantities, respectively.

Assuming perturbations are in the form

7 $$\psi \propto \psi (y){e}^{i(kx-\omega t)}$$

where *ω* is the frequency of the perturbation, k is the wave number.

Linearizing the Eq. (1), we get

8 $${\rho }^{0}\frac{\partial {\bf{V}}}{\partial t}=\frac{1}{c}{{\bf{J}}}^{0}\times {{\bf{B}}}^{1}+\frac{1}{c}{{\bf{J}}}^{1}\times {{\bf{B}}}^{0}+{\rho }^{1}{\bf{g}}-\nabla {p}^{1}$$

**z** · ∇× Eq. (8) yields

9 $$-\,i\omega (ik{\rho }^{0}{V}_{y}-\frac{\partial }{\partial y}({\rho }^{0}{V}_{x}))=-\,ik{\rho }^{1}g-\frac{1}{c}(\nabla \cdot {{\bf{J}}}^{1}){B}^{0}$$

where z is the unit vector in z direction.

From Eqs (5) and (6), we get

10 $$\nabla \cdot {{\bf{J}}}^{1}=-\,\frac{\partial {\rho }_{c}}{\partial t}=\frac{\partial {\rho }_{c}^{b}}{\partial t}=\frac{{\rm{1}}}{{\rm{4}}\pi }\nabla \cdot (\frac{\partial {\bf{E}}}{\partial t})$$

where *ρ*_*c*_ (${\rho }_{c}^{b}$) is the charge accumulation in (outside) the fluid element.

Substituting Eq. (10) in Eq. (9) we get

11 $$-\,i\omega (ik{\rho }^{0}{V}_{y}-\frac{\partial }{\partial y}({\rho }^{0}{V}_{x}))=-\,ik{\rho }^{1}g-\frac{1}{{\rm{4}}\pi c}\omega k{E}_{x}{B}^{0}.$$

Supposing that the plasma is incompressible

12 $$\nabla \cdot {\bf{V}}=0$$

we get the following equation

13 $${V}_{x}=\frac{i}{k}\frac{\partial {V}_{y}}{\partial y}.$$

From the linearized continuity equation

14 $$\frac{\partial {\rho }^{1}}{\partial t}+{\bf{V}}\cdot \nabla {\rho }^{0}=0$$

We get

15 $${\rho }^{1}=\frac{1}{iw}\frac{\partial {\rho }^{0}}{\partial y}{V}_{y}.$$

Form the relation *c***E** + **V** × **B** = 0 we get

16 $${E}_{x}=-\,\frac{1}{c}{V}_{y}{B}^{0}$$

Substituting the Eqs (13) and (16) into the Eq. (11), we get

17 $${\rho }^{0}\frac{{\partial }^{2}{V}_{y}}{\partial {y}^{2}}+\frac{\partial {\rho }^{0}}{\partial y}\frac{\partial {V}_{y}}{\partial y}-{k}^{2}({\rho }^{0}+\frac{g}{{\omega }^{2}}\frac{\partial {\rho }^{0}}{\partial y}-\frac{{\rm{1}}}{{\rm{4}}\pi }\frac{{B}^{{0}^{2}}}{{c}^{2}}){{\rm{V}}}_{{\rm{y}}}=0$$

Assume the initial plasma density has exponential dependence on *y*

18 $${\rho }^{0}(y)={\rho }^{0}{e}^{\frac{y}{s}}$$

where *s* is a constant, Eq. (17) can be written as

19 $$\frac{{\partial }^{2}{V}_{y}}{\partial {y}^{2}}+\frac{1}{s}\frac{\partial {V}_{y}}{\partial y}-{k}^{2}(1+\frac{g}{s{\omega }^{2}}-\frac{{V}_{A}^{2}}{{c}^{2}}){{\rm{V}}}_{{\rm{y}}}=0$$

where ${V}_{A}^{2}=\frac{{B}^{{0}^{2}}}{4\pi {\rho }^{0}}$ is the square of the nominal Alfvén speed^12^. Here ${V}_{A}=\frac{{B}^{{\rm{0}}}}{\sqrt{4\pi {\rho }^{0}}}$ is called nominal Alfvén speed because it is not the actual speed of the Alfvén wave^13,14^.

Assume the stratified plasma is bounded by two rigid boundaries *y* = *0* and *y* = *h*. The discrete solutions of Eq. (19) can be found of the form

20 $${V}_{y}(y)={{\rm{C}}}_{0}\,\sin \,(\frac{m\pi y}{h}){e}^{-\frac{y}{2s}}$$

where C_0_ is a constant.

Substituting the Eq. (20) into the Eq. (19), we get a general dispersion relation

21 $$\omega =\pm \,i{(\frac{g}{s}\frac{{h}^{2}{k}^{2}}{{h}^{2}{k}^{2}+{m}^{2}{\pi }^{2}+{h}^{2}/4{s}^{2}-{{V}_{A}}^{2}/{c}^{2}})}^{\frac{1}{2}}.$$

The full expression of the linear growth rate of MRTI is

22 $$\sigma ={(\frac{g}{s}\frac{{h}^{2}{k}^{2}}{{h}^{2}{k}^{2}+{m}^{2}{\pi }^{2}+{h}^{2}/4{s}^{2}-{{V}_{A}}^{2}/{c}^{2}})}^{\frac{1}{2}}.$$

To investigate the effect of charge accumulation on the linear growth rate of RTI, we nondimensionalize Eq. (21) with the following expressions

$$\sigma =-\,{\rm{i}}\omega \,{\sigma }^{\ast }=\sigma {({\omega }_{{\rm{pe}}})}^{-1}\,{g}^{\ast }=g{({\rm{s}}{\omega }_{pe}^{2})}^{-1}\,{k}^{\ast }=ks,\,{h}^{\ast }=h{(s)}^{-1}.$$

where *ω*_pe_ is the plasma frequency. We get

23 $${\sigma }^{\ast }=({g}^{\ast }\frac{{h}^{\ast 2}{k}^{\ast 2}}{{h}^{\ast 2}{k}^{\ast 2}+{m}^{2}{\pi }^{2}+{h}^{\ast 2}/4-{{V}_{A}}^{2}/{c}^{2}})$$

**Figure 2:**
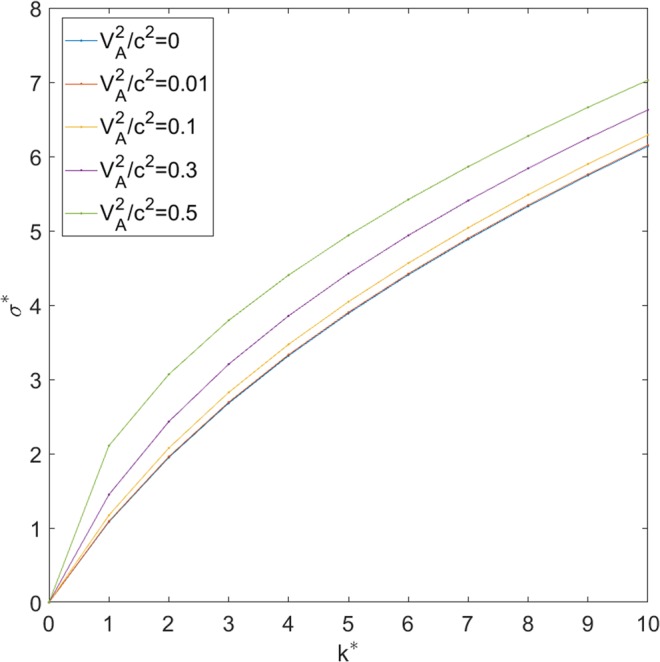
The growth rate of RTI (σ^*^) versus wave number (k^*^) for different values of ${V}_{A}^{2}/{{\rm{c}}}^{2}$. *V*_*A*_ and *c* are the nominal Alfvén speed and light speed, respectively.

Figure 2 shows the dimensionless dispersion relation for the configuration where h^*^ = 1, *m* = 1, g^*^ = 10, ${V}_{A}^{2}/{{\rm{c}}}^{2}$ = 0, 0.01, 0.1, 0.3, 0.5. Note that the curve representing ${V}_{A}^{2}/{{\rm{c}}}^{2}$ = 0.01 is basically coincided with that of ${V}_{A}^{2}/{{\rm{c}}}^{2}$ = 0. When *V*_*A*_ = 0, Eq. (21) represents the dispersion relation for the classical RTI^15^, and the growth rate is the same as that of the classical RTI. When *V*_*A*_ > 0, the growth rate is larger than that of the classical RTI, and increases with the increase of ${V}_{A}^{2}/{{\rm{c}}}^{2}$.

In planetary plasma, in general ${V}_{A}\ll c$, the growth rate is basically the same as that of the classical RTI. The contribution of charge accumulation to the growth of MRTI can be neglected. Ignore the displacement current term in Maxwell’s equations when calculate the linear growth rate of MRTI is acceptable. However, the physical description of the MRTI in planetary plasma which attribute the growth of MRTI to charge accumulation is wrong. The physical description of the growth of MRTI is similar to that of neutral fluid^16,17^. In laboratory plasma, the nominal Alfvén speed is not subject to the constraint ${V}_{A}\ll c$, the growth rate can be much larger than that of the classical RTI. Under such condition, the charge accumulation effect and the displacement current term cannot be ignored. However, the physical description in Fig. 1 which attribute the growth of MRTI to charge accumulation is not accurate, the mechanism for the growth of RTI in neutral fluid works too^16,17^. Also from Eq. (22) one can see that when the wave number k is very large, the linear growth rate reduce to that of classical RTI, and the effect of charge accumulation can be neglected.

Time scale analysis showed that everything involving charge separation happens on time scales of the inverse plasma frequency, and in such short time scales, the displacement current term cannot be ignored^18,19^. The MRTI process involves charge accumulation, so the displacement current term should not be ignored during the calculation of the growth rate of MRTI. The displacement current term is neglected because it’s generally small compared to ***J*** and curl ***B*** term. However, as we can see from Eq. (3) that the $\frac{\partial {\bf{E}}}{\partial t}$ has the same order of magnitude as **J**, and the changing electric field have significant effects on the dynamics of the plasma^20–22^. Ignore the displacement current term exclude the effects of electric field on the dynamics of the plasma. It is generally considered that the displacement current, which is closely related to charge separation, must be become negligible under the assumption of quasi-neutrality. However, studies show that when the nominal Alfvén speed is comparable to or larger than the light speed, the displacement current cannot be neglected, regardless of the quasi-neutrality considerations^16,23,24^, and this is consistent with our result discussed above.
